# Epigenetic Regulation of EMP/EMT-Dependent Fibrosis

**DOI:** 10.3390/ijms25052775

**Published:** 2024-02-28

**Authors:** Margherita Sisto, Sabrina Lisi

**Affiliations:** Department of Translational Biomedicine and Neuroscience (DiBraiN), Section of Human Anatomy and Histology, University of Bari, Piazza Giulio Cesare 1, I-70124 Bari, Italy; sabrina.lisi@uniba.it

**Keywords:** epigenetic, inflammation, fibrosis, DNA methylation, histone modification, ncRNA

## Abstract

Fibrosis represents a process characterized by excessive deposition of extracellular matrix (ECM) proteins. It often represents the evolution of pathological conditions, causes organ failure, and can, in extreme cases, compromise the functionality of organs to the point of causing death. In recent years, considerable efforts have been made to understand the molecular mechanisms underlying fibrotic evolution and to identify possible therapeutic strategies. Great interest has been aroused by the discovery of a molecular association between epithelial to mesenchymal plasticity (EMP), in particular epithelial to mesenchymal transition (EMT), and fibrogenesis, which has led to the identification of complex molecular mechanisms closely interconnected with each other, which could explain EMT-dependent fibrosis. However, the result remains unsatisfactory from a therapeutic point of view. In recent years, advances in epigenetics, based on chromatin remodeling through various histone modifications or through the intervention of non-coding RNAs (ncRNAs), have provided more information on the fibrotic process, and this could represent a promising path forward for the identification of innovative therapeutic strategies for organ fibrosis. In this review, we summarize current research on epigenetic mechanisms involved in organ fibrosis, with a focus on epigenetic regulation of EMP/EMT-dependent fibrosis.

## 1. Introduction

Fibrosis, characterized by the deposition of connective tissue in a tissue or organ, represents a reaction to an injury and has reparative or pathological significance. The fibrotic evolution of a tissue or organ can have very negative consequences, leading to the inability to perform normal physiological functions and resulting in a pathological condition with high mortality [1,2,3,4]. Fibrosis is often associated with pathologies characterized by a chronic inflammatory state, such as autoimmune diseases or tumors. In these circumstances, the prolonged release of growth factors and/or pro-inflammatory factors such as transforming growth factor-β (TGF-β) or various cytokines mediate the activation of a cellular transformation process called epithelial–mesenchymal plasticity (EMP) [5]. When EMP is activated, the epithelial cells, which have a phenotype of adherent cells closely connected to each other and are not invasive, become transformed, assuming a hybrid epithelial/mesenchymal phenotype and/or a completely mesenchymal phenotype. In this case, the process is defined as epithelial-to-mesenchymal transition (EMT) [5]. These cells acquire much higher migratory capabilities and are able to deposit extracellular matrix (ECM) proteins. The triggering of various cascades of molecular interconnected events leads to an exacerbation of the inflammatory state or to tumor proliferation and metastasis, with serious consequences [6,7,8]. Despite the fact that fibrosis appears to be a partly reversible process in various clinical studies [9], unfortunately, therapeutic options are still very limited. In recent years, very innovative studies have demonstrated how epigenetic modifications, by triggering or inhibiting gene transcription depending on the circumstances, can reprogram gene expression by adapting it to exposure to various risk factors [10]. This has been demonstrated, for example, in idiopathic pulmonary fibrosis (IPF) or in patients with non-alcoholic fatty liver disease, in which biopsy samples show higher expression of DNA methyltransferase, suggesting that DNA methylation could represent a predisposing factor for the onset of these pathologies [11]. The application of sequencing technology has demonstrated that the activation of fibroblasts, involved in collagen deposition during fibrogenesis, depends on various epigenetic modifications affecting the DNA to be transcribed [12]. Furthermore, epigenetic modifications appear to be largely involved in the modifications of epithelial cells towards the mesenchymal phenotype, a process essentially mediated by EMT [13,14].

Some authors have described in detail the mechanisms through which the main epigenetic modifications would act, inducing a regulation of the fibrotic evolution of the inflammatory processes and determining the transcription of pro-fibrotic genes [12]. In addition, epigenetics could explain the reversibility of the fibrosis [10]. However, although recent discoveries tend toward the involvement of epigenetic modifications in EMP/EMT-dependent fibrosis, the elucidation of the mechanisms involved still seems far from clear. This review aims to collect the latest discoveries made by studying the involvement of epigenetic modifications in the activation of EMP/EMT-dependent fibrosis, with the aim of suggesting new therapeutic perspectives.

## 2. The Dynamic Balance between EMT and EMP

**Figure 1:**
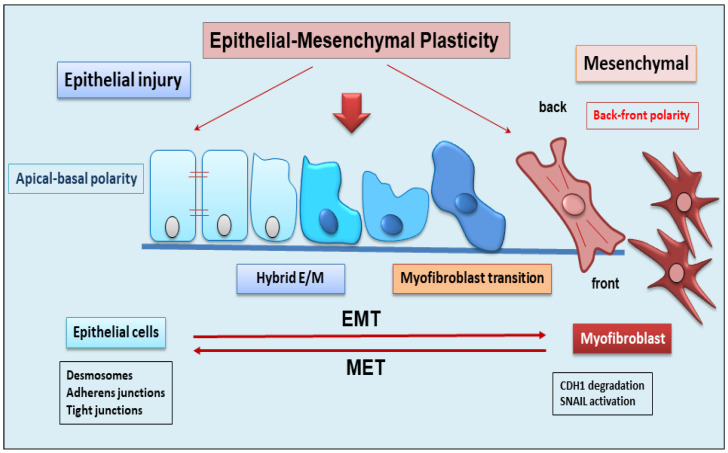
Schematic representation of epithelial to mesenchymal plasticity (EMP). EMP, in response to epithelial injury, allows cells to convert between multiple states across the epithelial to mesenchymal transformation, acquiring hybrid epithelial/mesenchymal phenotypic features.

EMT is a dynamic complex process during which epithelial cells reduce their epithelial properties, gradually dissolving cell–cell junctions and rebuilding cell–matrix connections to acquire characteristics typical of mesenchymal cells [15,16,17]. When the mechanism of EMT was identified, it was discovered that EMT was responsible for multiple processes involved in embryonic development, such as gastrulation, neural crest formation, and heart development [15,18]. But researchers soon demonstrated that the activation of EMT also affected physiological processes represented by wound healing [19], with the fibrotic evolution of diseases characterized by chronic inflammation, and with the formation of metastases from primary tumors. EMT is classified into three functional types: type I, involved in embryonic morphogenesis; type II, responsible for normal wound healing, but this type can enhance myofibroblast activation leading to the deposition of high levels of ECM proteins and fibrosis in chronic diseases; and type III, characteristic of malignant epithelial cells that acquire a migratory phenotype capable of invading and metastasizing [20,21,22]. EMT is a reversible phenomenon, and the resulting cells shift back from motile, multipolar mesenchymal types to polarized epithelial types via the mesenchymal–epithelial transition (MET) process [23]. Therefore, until now, EMT was considered as an “all or nothing” program wherein the cells can exist with an epithelial morphology or in a mesenchymal state. Interestingly, novel insights have shown that the cells that undergo to EMT present multiple intermediate phenotypes. This new concept, recently named as EMP, defines the capacity of the cells to interconvert between several states along the epithelial–mesenchymal spectrum, thereby acquiring hybrid epithelial/mesenchymal phenotypic features [24,25]. Intriguingly, this cellular plasticity is very pliable, and epithelial cells often undergo partial reorganization and combine epithelial and mesenchymal features following the EMT process [26] (Figure 1). Indeed, such cellular shifts and the resultant heterogeneity provide the cells with the flexibility to face different physiological (embryonic development, wound healing) and pathological (organ fibrosis, cancer) conditions [27]. The dynamics of EMT/EMP and MET are controlled by a complex network of transcription factors (TFs) [28]. TFs, in epithelial cells, determine the transcription of a variety of genes involved in the activation of EMT programs [29]. These changes in transcription, sometimes seen as gene reprogramming, involve three TFs families: *Snail* (*Snail1*) and *Slug/Snail2*, *ZEB1* and *ZEB2*, and *Twist* [28,30]. All of these TFs share the ability to repress epithelial genes like the E-cadherin encoding gene *CDH1* via binding to E-Box motifs in their cognate promoter regions [15]. In parallel, the EMT-TFs, directly or indirectly, activate genes associated with a mesenchymal phenotype, including *VIM* (Vimentin), *FN1* (Fibronectin), and *CDH2* (N-cadherin) [15,28]. Upon induction of an epithelial plasticity response, they are considered as “master” drivers of the EMT program, conferring cellular shift among the epithelial–mesenchymal spectrum [26,29]. Interestingly, with increasing data relating to the mechanisms of activation of EMT pathways, other than the signaling molecules regulating EMT, it becomes clear that activation and execution of EMT occur as a result of genetic and epigenetic processes. The study of epigenetic regulation is an important aspect of modulation of EMT [25,31], and various epigenetic mechanisms appear to be involved in the modulation of EMT, although it is still difficult to correlate all of the scientific data collected together [25]. Currently, most of the studies carried out concern the epigenetic control of EMT during cancer progression and metastases formation [16,17,18]. Similarly, recent discoveries have also attributed a key role to epigenetic modifications in the activation of the EMP process. Numerous pieces of evidence have demonstrated an altered expression of the main epigenetic modifications underlying the delicate balance between EMP and EMT, including histone modification, DNA methylation, and non-coding RNA, which could facilitate cancer metastasis [31].

## 3. Role of EMP/EMT in Organ Fibrosis

In recent years, our knowledge of the fibrotic process has been remarkably increased by the characterization of cellular mediators, key inflammatory and profibrogenic cytokines, molecular factors, and the evolution of new pathogenetic scenarios. A major determinant of fibrosis is the continuous spread of fibroblasts and myofibroblasts, which suggests the question of how this cellular system can be fed [32]. Experiments conducted in the last 2 years have shown that cellular plasticity, which also includes the phenomenon of EMP, is not limited exclusively to development; it also characterizes cells that undergo reprogramming that occurs during the repair of tissue damage, during fibrotic processes, and during carcinogenesis [26]. However, knowledge of the molecular mechanisms involved in the cell’s ability to modify its phenotype by evolving into another cell type is still at the beginning. The mechanisms underlying EMT are much more explored and known, and numerous studies have been conducted to evaluate the key role of EMT in fibrosis. In the context of identifying cellular drivers of fibrosis, various in vitro and in vivo studies have reported that EMT is a key mechanism during fibrogenesis, substantially contributing to the increase in interstitial fibroblasts and myofibroblasts, and interrupting its progression can have a profound impact on the onset and progression of related diseases, particularly fibrosis [21,33,34]. In fact, a fibrotic evolution of chronic diseases can lead to pathological states affecting various organs, including the lungs, liver, kidneys, heart, and salivary glands [35].The following paragraph summarizes the most recent discoveries derived from evaluating the molecular mechanisms underlying the process of EMT-dependent fibrosis in various organs in pathological conditions in order to identify potential therapeutic targets.

### Contribution of Epithelium to the Fibrotic Organ Process via EMT Activation

In the last few years, important findings have demonstrated that liver epithelial cells undergo the EMT process, contributing to their transformation into myofibroblasts. Indeed, hepatocytes in which the *Snail1* gene was deleted by using Cre-loxP technology showed a reduction in EMT factors and a decrease in the severity of the inflammatory response compared to controls [36]. Moreover, Rowe et al. have examined a panel of genes known to contribute to the progression of liver fibrosis, including interstitial collagen types I and III and fibroblast markers, demonstrating that *Snail1* determines an increased expression of profibrotic genes such as those encoding for type I or type II collagen, vimentin, and *FSP1* in the liver [36]. These data demonstrate that the hepatocyte Snail1 gene is a potent inducer in the progression of hepatic fibrosis. Indeed, explorations have been performed to determine the origin of hepatic myofibroblasts activated in response to the type of liver injury. In particular, hepatic stellate cells (HSCs) are capable of transforming into contractile myofibroblasts after liver injury. In a mouse model subjected to hepatotoxic CCL_4_ liver injury, activated HSCs transformed almost totally into myofibroblasts, whereas cholestatic bile duct ligation treatment preferentially stimulated portal fibroblasts [37,38]. It is interesting to underline that HSCs, when not activated, predominantly express epithelial markers compared to mesenchymal ones, and, following damage, can become activated and undergo a change in phenotype driven by EMT [39].

Nowadays, it has been experimentally proven that the activation of an EMT program occurs in a variety of pulmonary fibrosis diseases [40]. A study highlighted the contribution of the bronchial epithelial cells that, when treated with TGF-β1, are able to acquire myofibroblast phenotypes, thereby leading to peribronchial fibrosis. This process drives airway epithelium remodeling, which is a feature of asthma [41]. In IPF, alveolar epithelial cells undergo EMT, inducing the formation of fibroblastic foci and thus triggering the fibrotic destruction of the lung architecture [42,43]. Interestingly, pleural mesothelial cells also undergo a special type of EMT, mesothelial-to-mesenchymal transition (MMT), during IPF pathogenesis. In the MMT process, the mesothelial cells, during serosal inflammation, acquire the mesenchymal phenotype and complete their transformation into myofibroblasts, thus contributing to the progression of parenchymal fibrosis that results in a progressive decline in lung function [44]. Indeed, an important report demonstrated the presence of pleural mesothelial cells exhibiting mesenchymal markers in the lung parenchyma of patients with IPF after fibrogenic stimulation in vivo and a correlation between disease severity and the degree of fibrosis [44].

It has now been widely demonstrated that tubular epithelial cells (TECs) are involved in a process of EMT-dependent fibrosis in chronic renal failure [45]. However, the percentage of TECs that transform into myofibroblasts during this process is not yet known. This has led to the proposal that renal epithelial cells would undergo a partial EMT (pEMT), resulting in renal fibrotic evolution [46,47]. During pEMT, TECs maintain some characteristics of epithelial cells and show, at the same time, typical markers of fibroblasts, acquiring an intermediate phenotype between the two cell types [46,47]. Therefore, TECs in this partial mesenchymal and epithelial phenotype remain attached to the basement membrane during the fibrotic process. Recently, it was demonstrated that Snail1 is able to trigger the pEMT process in TECs, relaying crucial signals for fibrogenic cytokine release and promoting differentiation into myofibroblasts, thus contributing to the exacerbation of the inflammatory response [46].

EMT is also activated in inflammatory bowel diseases (IBDs) such as ulcerative colitis (UC) and Crohn’s disease (CD) [48]. In IBD patients, persistent intestinal inflammatory factors injure intestinal epithelial cells, determining reparative reactions that lead to the triggering of the EMT process and perpetuating a severe fibrotic condition [48,49]. Confirming this, the presence of high levels of tumor necrosis factor-like ligand 1A (TL1A) in the intestinal specimens of patients with UC and CD has been detected, which represents a potent inducer of EMT in intestinal fibrosis. As expected, the TGF-β1/Smad3 pathway may be involved in TL1A-induced EMT [49,50,51].

Recent discoveries have also shown that uncontrolled fibrosis is present in the heart and is triggered by EMT and its special type, endothelial to mesenchymal transition (EndMT) [52,53]. Epicardial EMT is activated after myocardial infarction, atherosclerosis, and valve dysfunction, and it determines angiogenesis and healing [54]. Under TGF-β stimuli, cardiac fibroblasts transdifferentiate into myofibroblasts, acquiring a phenotype similar to that of smooth muscle cells. Furthermore, fibroblasts can also originate from endothelial cells through EndMT, giving rise to the progression of cardiac fibrosis [54]. In addition, TGF-β-driven EMT responsible for cardiac fibroblast formation appears to be triggered by the Hippo pathway, an evolutionarily conserved kinase cascade [55], as seen in recent in vitro findings [56]. In addition, the process of cardiac fibrosis seems to be regulated by C-Ski protein, identified as an inhibitory regulator of TGF-β signaling [57].

A flourishing research field is focused on the evaluation of the pathways involved in the fibrotic process observed in the salivary glands (SGs) derived from Sjögren’s Syndrome (SS) patients. Fibrogenesis observed in SGs can be considered the end result of chronic, intense inflammatory reactions induced by a variety of stimuli in this autoimmune disease [7,8,58,59]. Pioneering studies aimed at correlating SS with a fibrotic evolution of the salivary glands were conducted over 10 years ago, demonstrating a significant association between stimulated salivary flow, the focus score, and fibrosis in a high number of SS biopsy specimens. In comparison, unstimulated salivary flow appears to be weakly associated with the focus score and is not always correlated with fibrosis, which was considered an excellent measure of irreversible damage [58]. In all cases, SG fibrosis is linked with an evident impairment of organ function that leads to progressive atrophy and a decrease in quality of life for patients [60].

In this context, using technology to create transgenic mice that conditionally overexpress active TGF-β1, experimental data have confirmed that the overexpression of active TGF-β1 leads to an abnormal accumulation of ECM proteins and severe hyposalivation and acinar atrophy in the mutated mice [61]. More recently, studies have demonstrated an exuberant upregulation of TGF-β1 in SS SGs, which induces the loss of epithelial features and the acquisition of mesenchymal features in SG epithelial cells via triggering of the EMT program through the TGF-β1/Smad/Snail signaling pathway [62]. Indeed, TGF-β1 seems to be able to regulate EMT through both main pathways: the canonical Smad-dependent and non-canonical Smad-independent signaling pathways [7,8,62].

## 4. Main Epigenetic Mechanisms

Each phase of gene expression can undergo epigenetic modifications, thus leading to the synthesis or inhibition of certain downstream proteins. The epigenetic processes involve DNA methylation, histone modification, chromatin remodeling, and the effects of noncoding RNA.

The phenomenon of DNA methylation is an essential process for the physiological development of the individual and plays a key role in processes widely studied in recent years, such as genomic imprinting. DNA methylation and demethylation represent heritable epigenetic signatures that are evolutionarily conserved and do not involve an alteration of the DNA sequence but can, however, lead to widely modified gene expression [63,64].

Methylation of DNA is an epigenetic mechanism that consists of the transfer of a methyl group from *S*-adenyl methionine (SAM) to the C-5 position of a cytosine residue in a dinucleotide CG or polynucleotide CGGCGG context, also termed CpG islands, to form 5-methylcytosine catalyzed by DNA methyltransferases (DNMTs) [65]. The DNMT family comprises various elements: DNMT1, DNMT2, DNMT3A, DNMT3B, and DNMT3L [66]. Notably, the methylation of the promoter region or gene has different effects; excess promoter methylation silences the gene, while a reduction in promoter methylation causes increased gene expression [67]. On the contrary, at the gene level, an excess of methylation determines active transcription of the gene itself, while the methylation of the gene has a meaning that is not yet well known [68].

Similar to DNA methylation, post-translational histone modifications do not affect the DNA nucleotide sequence but alter its accessibility to the transcriptional machinery. Histones are small basic proteins assembled into nucleosomes and are essential to compact and stabilize DNA by making the DNA sites implicated in gene transcription accessible [69]. Each nucleosome is composed of approximately 150 base pairs of DNA and two copies of the four core histones: H2A, H2B, H3, and H4 [69]. In addition, H1 protein acts as a linker histone-compacting chromatin, and its role is to stabilize the internucleosomal DNA but does not form part of the nucleosome. Histone proteins, through post-translational modifications, control chromatin structure, triggering the transition from open chromatin, called euchromatin, which is actively transcribed, to a compacted chromatin structure called heterochromatin. In this compact form, DNA is not accessible to transcriptional machinery and thus cannot be transcribed, resulting in gene silencing [70].

Several of the best-known post-translational modifications of histones include acetylation, methylation, phosphorylation, and ubiquitylation. However, in recent years, other histone modifications have been identified, such as GlcNAcylation, citrullination, krotonylation, and isomerization, which still need to be further explored [71].

Acetylation modulates transcriptional activity through the neutralization of the positive charge present on the lysine residues of histone proteins. This action has the potential to weaken the interactions between histones and DNA, making them less stable, thus allowing gene transcription [72,73]. Acetylation consists of the addition of acetyl groups to lysine residues, neutralizing their positive charge. Thus, acetylation induces and enhances gene expression. Histone acetylation and deacetylation are catalyzed by histone acetyltransferases (HATs) and histone deacetylases (HDACs), respectively.

HDACs remove acetyl groups from acetylated proteins, consequently repressing gene expression. They are classified into four classes: class 1 (HDAC1,2,3,8), class 2 (2a: HDAC4,5,7,9; 2b: HDAC6,10), class 3 (SIRT), and class 4 (HDAC11). Therefore, sirtuin proteins, classified within class III HDACs, require nicotinamide adenine dinucleotide (NAD) as a cofactor for their catalytic activity. To date, 18 mammalian HDACs have been identified and classified into the above different classes [74].

Methylation occurs in both the lysine and arginine residues of histones H3 and H4 and, in particular, does not alter the charge of the histone protein. Arginine methylation, which requires arginine methyltransferase activity, induces gene transcription, while lysine methylation, which requires histone methyltransferase, can have either a positive or negative effect on transcription due to the site involved in the methylation [75,76,77,78]. Recently, it has been demonstrated that histone methylation is also a reversible event through the mechanism of histone demethylases [79].

Phosphorylation influences all core histones, with several effects on each. Phosphorylation of serine residues 10 and 28 of histone H3 and serine residue T120 of histone H2A is involved in chromatin condensation through the phases of cell replication during mitosis. Phosphorylation of the S139 residue in histone H2A evidences a landing point for the interaction with factors involved in the repair of DNA damage [80]. However, phosphorylation of histone H2B is not as well known but appears to induce chromatin compaction through several mechanisms such as apoptosis, DNA fragmentation, and cell necrosis [81].

All histone proteins can undergo a mechanism of ubiquitylation; however, in the last few years, studies have highlighted two well-characterized proteins, H2A and H2B, which are most frequently ubiquitinated in the nucleus [82]. Histone ubiquitination is linked with the activation of gene expression, but many studies have demonstrated that the presence of a single ubiquitin has different effects on H2A and H2B. Indeed, mono-ubiquitylated H2A is linked with gene silencing, while if the interaction concerns H2B, transcription activation is induced [82].

Epigenetic regulation also involves actively non-coding RNA (ncRNAs) and it has been widely discovered that ncRNAs are able to modulate gene expression at both transcriptional and post-transcriptional levels. ncRNA refers to a functional RNA molecule that is transcribed from DNA but is not translated into a protein [83]. NcRNA are divided into two broad categories based on their length: short ncRNAs, with a number of nucleotides less than 30, and long ncRNAs (lncRNAs), which include those RNAs with a number of nucleotides greater than 200 [83]. The three main classes of short noncoding RNAs include microRNAs (miRNAs), short interfering RNAs (siRNAs), and piwi-interacting RNAs (piRNAs).

MiRNAs and DNA methylation are the two epigenetic events that have emerged in recent years and correlate to the modulation of gene expression [84]. Notably, miRNAs act by linking to a specific target messenger RNA through a complementary sequence; this binding determines the fragmentation and degradation of the mRNA, consequently blocking the translation event. Interestingly, the presence of a mutual regulation between miRNAs and DNA methylation has been shown in human tumors. Indeed, miRNAs modulate DNA methylation by acting on the transcription of genes implicated in the synthesis of DNA methyltransferases [85].

**Figure 2:**
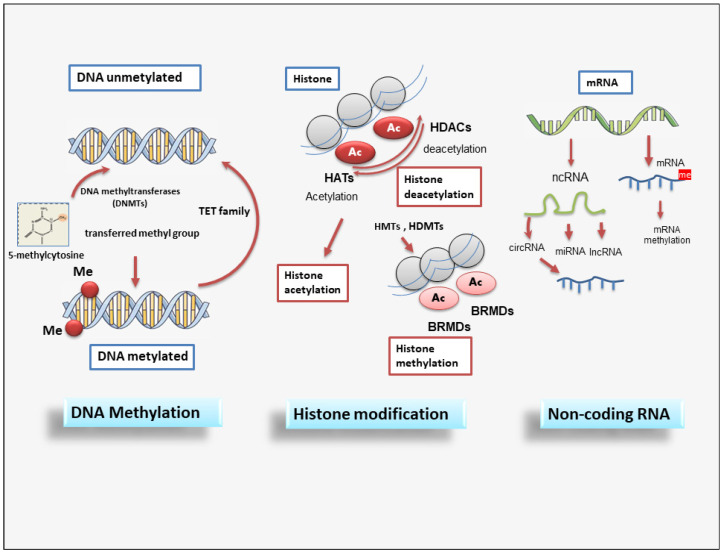
A schematic overview of epigenetic modifications. Epigenetic regulation involves DNA methylation, histone modification, and non-coding RNAs. DNMT family members mediate DNA methylation, which suppresses gene transcription by adding a methyl group to the cytosine position. Histone methylation is catalyzed by HMTs and HDMTs, and histone acetylation is regulated by HATs and HDACs. Non-coding RNAs include miRNAs, circRNAs, and lncRNAs. BET (Bromodomain and extraterminal); BRMDs (bromodomains); DNMT (DNA methyltransferase); HATs (Histone acetylases); HDACs (Histone deacetylases); HDMTs (Histone demethylases); HMTs (Histone methyltransferases); lncRNA (long non-coding RNA); TET (Ten-eleven translocation).

SiRNAs represent small RNA molecules whose function is to repress the expression of a gene by binding to the mRNA, inducing its degradation, and thus preventing post-transcriptional gene and subsequent protein synthesis [85]. SiRNAs are gained from a long double-stranded RNA molecule that is cut into many small fragments by Dicer endoribonuclease. The siRNAs obtained are added to the so-called RISC complex (RNA-induced silencing complex) to form the inactive RISC-siRNA complex. Once activated, the siRNA loses one of the two strands and binds to the mRNA target messenger, i.e., the mRNA whose translation into protein is to be prevented [86,87]. The piRNAs are a complex class of sncRNAs that specifically interact with the PIWI protein subfamily of the Argonaute family [88]. Current research evidences that this interaction between PIWI proteins and piRNAs regulates novel epigenetic mechanisms such as DNA rearrangements; however, this has yet to be clarified. Recently, lncRNAs were discovered as important regulators of the epigenetic status of the human genome. LncRNAs are RNA fragments longer than 200 nucleotides that have various activities, such as chromatin remodeling and transcriptional and post-transcriptional regulation, and act as precursors of siRNAs with the function of gene silencing [89,90,91]. Many lncRNAs form complexes with proteins, leading to modifications in the conformation of chromatin [92,93]. In addition, a novel member of the lncRNA class, circular RNAs (circRNAs), are characterized by a covalently closed loop. They are recognized to have distinct biogenesis and to regulate gene expression and biological processes through different mechanisms, with some miRNA-sponging circRNAs identified [94]. A schematic overview of epigenetic modifications is reported in Figure 2.

## 5. Epigenetics Regulation of EMP/EMT-Dependent Fibrosis

The studies conducted in order to evaluate a possible connection between epigenetics, EMP, and fibrosis are leading to the first discoveries, for example, in airway persistent inflammation [95], but require further investigation. On the contrary, the field of studies conducted on the role of epigenetic modifications in EMT-dependent fibrosis is much more flourishing. One of the hot topics in the last few years has been the association between epigenetic regulation and fibrotic processes triggered by EMT [13]. Epigenetic modulation of tissue–stroma interactions involves several types of alterations that have been shown in recent years to play a determining role in the activation of the EMT program and the consequent EMT-dependent fibrosis [13].The following paragraphs illustrate the current knowledge concerning the epigenetic aberrations involved in the fibrotic evolution induced by EMT during pathological processes.

### 5.1. DNA Methylation in EMT-Dependent Fibrosis

DNA methylation, catalyzed by DNMTs, represents one of the best-represented mechanisms of epigenetic control and modulation in EMT [96]. It is known that the downregulation of E-cadherin expression is fundamental to the evolution of the EMT process [97]. DNA methylation, regulated by DNMTs, seems to affect *CDH1* expression. The ten-eleven translocation (TETs) family, instead, is implicated in *CDH1* demethylation [98]. Transformations in *CDH1* promoter methylation lead to diminished E-cadherin protein expression in several fibrotic diseases [99,100,101]. Direct methylation of transcription factors by DNMT1 concurs with EMT program activation in renal epithelial cells [102] and, as expected, DNA methylation inhibition through specific inhibitors reversed EMT in arsenic-triggered renal fibrosis [103].

Therefore, interesting studies have evidenced the essential role of DNMTs in cardiac fibrosis, resulting in the activation of the EndMT process. The inhibition of the suppressor of cytokine signaling 3 (*SOCS3*) mediated by DNMT1 determines the activation of STAT3 that induces cardiac fibroblast activation and collagen deposition in cardiac fibrosis [104]. This phenomenon was also detected in the fibrotic skin of patients with systemic sclerosis, in which TGF-β induced the expression of DNMT3A and DNMT1 in fibroblasts in a SMAD-dependent manner, leading to a decreased expression of SOCS3 and facilitating activation of STAT3 to promote fibroblast-to-myofibroblast transition, collagen release, and fibrosis [104].

The transcriptional regulation driven by the DNA methylation pattern plays a pivotal role in liver fibrosis [10]. In particular, DNA methylation is involved in the differentiation of HSC during hepatic diseases characterized by a severe and progressive fibrotic process. These data were confirmed by downregulating *DNMT3a* and *DNMT3b* gene expression through the use of siRNA; in this case, DNA methylation was decreased, and HSC activation was subsequently suppressed [105]. Beyond DNMTs, another intriguing protein implicated in liver DNA methylation processes is glycine N-methyltransferase (GNMT). GNMT is the most abundant methyltransferase in the liver and hepatocytes. GNMT influences epigenetic regulatory determinants by competing with DNMT to regulate transmethylation flux [105]. The triggering of EMT in the liver appears to be related to activation by the hedgehog (Hh) pathway. Patched1 (PTCH1), a factor that negatively regulates Hh, is downregulated during the process of liver fibrosis and this appears to be related to its hypermethylation state. Recent studies have established the antifibrotic efficacy of salvianolic acid B (Sal B), attributing it to its ability to inhibit Hh-mediated EMT. An upregulation of *PTCH1* was noted due to a decrease in DNA methylation thanks to the inhibition of DNMT1. Interestingly, the increase in miR-152 in Sal B-treated cells was responsible for the hypomethylation of *PTCH1* by Sal B, and DNMT1 was found to be a direct target of miR-152 [106].

**Figure 3:**
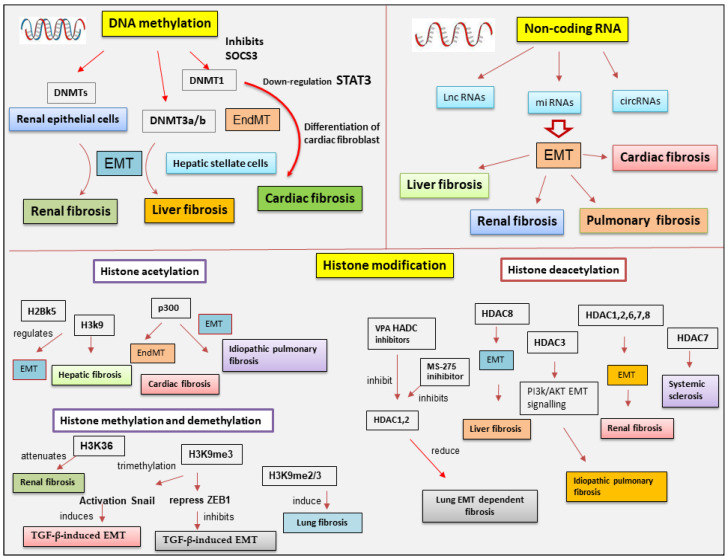
Scheme of the epigenetic modifications in EMP/EMT-dependent fibrotic diseases. DNMT (DNA methyltransferase); HAT (Histone acetylase); HDAC (Histone deacetylase); EndMT (Endothelial–mesenchymal transition); EMT (Epithelial–mesenchymal transition); SOCS3 (cytokine signaling 3); VAP (Valproic acid).

Several studies have mentioned altered DNA methylation linked to the evolution of chronic obstructive pulmonary disease and pulmonary fibrosis [104,107]. In addition, the effects of DNA methylation were linked to histone modifications and miRNA activity to induce or block gene expression in fibrotic progression [104,107] (Figure 3). An interesting study conducted on epithelial *cells* isolated from normal human bronchial epithelium exposed to nickel (NiCl2) demonstrated that, through the activation of the TLR4 signaling pathway and EMT, nickel is associated with the development of many chronic lung diseases, including pulmonary fibrosis [108]. It was experimentally demonstrated that NiCl2 exposure determines E-cadherin downregulation in normal bronchial epithelial cells associated with E-cadherin promoter DNA hypermethylation [109].

### 5.2. The Involvement of Histone Modifications in EMT-Dependent Fibrosis

#### 5.2.1. Histone Acetylation and Deacetylation

Histone acetylation and deacetylation are widely analyzed histone modifications and have been recently linked to the activation of the EMT program in cancer and fibrosis [72]. In fact, tumors with stem cell features propagate and determine far away metastases by triggering the advancing EMT program. Recent studies have demonstrated that acetylation of histone H2BK5 is crucial in the control of EMT [14]. For example, in trophoblast stem cells, H2BK5 acetylation influences the expression of key genes implicated in the conservation of epithelial characteristics. These trophoblast stem cells share similar H2BK5 acetylation-regulated gene expression when compared with stem-like claudin-low breast cancer cells, thus linking EMT-dependent development and EMT observed in cancer cells [14]. In particular, HATs regulate gene silencing or transcription through the modulation of the acetylation of histones, thus orchestrating gene expression to induce liver fibrosis [110,111]. Additionally, histone acetylation has been implied in pulmonary and cardiac fibrosis [10], performed through the activity of p300 HAT [112,113]. An interesting discovery enriched the molecular scenario by demonstrating that p300 HAT induces the fibrotic process in IPF and cardiac fibrosis via EMT and EndMT, respectively [114].

HDACs, firstly identified in liver fibrosis, can enhance the cellular migration and ECM deposition by myofibroblasts [115,116]. HDACs belonging to each class appear to be implicated in EMT-dependent fibrosis activation, as reported in the following subparagraphs.

##### Class I Histone Deacetylase Involvement in EMT-Dependent Fibrosis

Histone deacetylation is largely studied in pulmonary fibrosis. Epigenetic histone modifications through deacetylation could explain the persistently activated state of IPF fibroblasts [117], which indicated a “cancer-like” upregulation. According to this hypothesis, almost all class I (and class II) HDAC enzymes result in overexpression and this could be responsible for the abnormal repression of pro-apoptotic genes [117,118]. Under conditions of hypoxia, HDAC3 combines with WD repeat domain 5 (WDR5) to recruit histone methyltransferase, leading to decreased acetylation of H3K4 and to an increased methylated form of H3K4 [119].

Interesting studies also concern the use of specific HDAC inhibitors. The short-chain fatty acid valproic acid (VPA), a class I-specific HDAC inhibitor, was able to reduce lung EMT-dependent fibrosis in bleomycin (BLM)-treated mice with Smad2/3 deactivation [120]. VPA seems to work without Akt cellular pathway deactivation, presumably due to the fact that VPA specifically inhibits the activities of HDAC1 and HDAC2 [121,122] but not of HDAC3 (directly involved in PI3K/Akt EMT signaling) [123]. Confirming this, the Class I HDAC inhibitor entinostat (MS-275), specific for HDAC1 and HDAC3, determines the inactivation of the PI3K/Akt pathway in TGF-β-stimulated lung fibroblasts [123]. Entinostat suppresses the TGF-β-induced expression of SPARC, a matricellular protein involved in the ECM turnover and apoptosis resistance of lung myofibroblasts restoring the expression of SPRC’s negative regulator named ARHGEF3 (Rho guanine nucleotide exchange factor 3, also known as XPLN = exchange factor found in platelets and leukemic and neuronal tissues [124].

Recent data showed that HDAC3 inhibition results in the acetylation and degradation of a vector expressing the NOTCH1 intracellular domain (NICD1), thereby alleviating IPF [125]. This suggest that HDACs can deacetylate also non-histone targets with important consequences from the point of view of activated pathological mechanisms [126].

In addition, HDAC3 was upregulated in alveolar epithelial type 2 (AT2) cells from patients with IPF, and in AT2 cells from mice with BLM-induced pulmonary fibrosis. Moreover, HDAC3 deficiency in AT2 cells prevented mice from developing BLM-induced pulmonary fibrosis, characterized by a marked reduction of EMT in AT2 cells. In terms of mechanisms, we found that TGF-β1/SMAD3 can directly promote HDAC3 transcription and further inhibit GATA3 acetylation, thus promoting EMT in AT2 cells. GATA3 is a transcription factor and the most frequently mutated genes in breast cancer [127].

Recently, Chen et al. reported that inhibition of HDAC3 and Nuclear Factor Erythroid-Derived 2-Related Factor-2 (Nrf2) mitigates pulmonary fibrosis [128], and Zheng et al. suggested that HDAC3 accelerates pulmonary fibrosis by promoting EMT and inflammation through the Notch1 or STAT1 signaling pathway [72]. Actually, however, the main activator of EMT dependent fibrosis in lung is hypoxia that activate a signaling cascade that involves the activation of the transcription factor Snail [31]. HDAC3 significantly increased Snail expression under hypoxic conditions, and this effect was prevented by inhibition of Hypoxia-inducible factor 1-alpha (HIF1 α), a subunit of a heterodimeric transcription factor hypoxia-inducible factor 1 (*HIF-1*). HDAC3 increases the transcriptional activity of HIF-1α by promoting the binding of HIF-1α to the hypoxia-responsive element (HRE) sites of genes [129]. Since fibroblast migration is considered a critical contributor to lung fibrosis, it was recently demonstrated that HDAC3-miR224- Forkhead Box A1 (FOXA1) axis effectively regulated the migration and invasion of fibroblast cells under hypoxia [129]. The effective involvement of HDAC3 in EMT-dependent fibrosis in lung was demonstrated by the use of *HDAC3* siRNA that alleviated BLM-induced pulmonary fibrosis in mice. In addition, *FOXA1* gene was identified as the target gene of miR-224 in HDAC3-mediated alveolar EMT [129] and HDAC3 promotes hypoxia-induced alveolar EMT through stabilization of HIF-1α via the AKT pathway [129].

Interesting data were collected also for renal fibrosis. In human mesangial cells (HMC) treated with poly IgA1, HDAC1, HDAC2, and HDAC8 were upregulated, determining the subsequent activation of TGF-β/Smad2/3 and Jak2/Stat3 signalling pathways. These pathways activation leads to the proliferation of HMCs and facilitates ECM deposition and fibrosis progression [130]. Recent studies have used various HDAC inhibitors to evaluate the effects on EMT-related kidney fibrosis, demonstrating that class I HDAC inhibitors are more effective than class II HDAC inhibitors in regulating this process. For example, using specific siRNAs against HDAC1, 2, 3 and 8, it was seen that the knockdown of HDAC1, HDAC2 or HDAC3 did not hinder the expression of ECMs and the initiation of TGF-β1-dependent EMT. This result seems to be due to a compensatory mechanism that intervenes when one HDAC does not function and which activates the others more [131]. Indeed, HDAC1 and HDAC2 show high amino acid homology and compensatory functions between them [132]. Using UUO mice as a model of renal fibrosis, the efficacy of another selective inhibitor of class I HDACs called FK228 was evaluated in EMT-dependent fibrosis [133]. Rat renal interstitial fibroblasts and renal tubular epithelial cells were treated in vitro with TGF-β1, in the presence or not of the inhibitor FK228. The results indicated that FK228 is able to reduce ECM protein deposition in both in vivo and in vitro. FK228 also blocked the activation and proliferation of renal fibroblasts and led to increased acetylation of histone H3 and seems to suppress renal interstitial fibrosis via canonical-Smad- and non-Smad-EMT pathways [133]. 

In the same experimental model UUO mice, Chen et al. [134] demonstrated a correlation between the high expression of HDAC3 in renal fibrotic tissues, and the low expression of the klotho protein, a membrane-bound protein that acts as a permissive co-receptor for Fibroblast Growth Facrot (FGF)-23. Silencing of the *HDAC3* gene has no effect on Klotho expression; on the contrary, inhibiting the TGF-beta receptor through the use of the specific inhibitor SB431542 led to a slowdown of the renal fibrotic process. By performing a more targeted inhibition using a specific inhibitor of SMAD3, SIS3, it was demonstrated that the TGF-β/Smad3 pathway led to an upregulation of HDAC3 [134]. In addition, it was been demonstrated that HDAC3 forms a complex with NCoR and NF-κB that acts on the klotho promoter, giving rise to a EMT-mediated pro-fibrotic renal transduction cascade that initiates from TGF-β and has HDAC3 as an intermediary [134].

A role of the complex formed by HDAC3 and Smad2/3 and NcoR in renal fibrosis has also been demonstrated in patients with focal segmental glomerulosclerosis who show high levels of elevated HDAC3. The complex appears to have an inhibitory effect on the miR-30d promoter, promoting renal fibrosis [135], although a concomitant activation of EMT in this fibrotic process has not yet been demonstrated.

HDAC3 also appears to regulate the expression of TIMAP protein (membrane-associated protein and inhibited by TGF-β). TIMAP expression is reduced in mice with renal fibrosis, probably due to the overexpression of TGF-β. The high concentration of TGF-β in fibrotic tissues would determine an overexpression of HDAC3 with the consequent activation of the TGF-β-HDAC3/Smad-TIMAP pathway which leads to a reduction in the expression of TIMAP [136]. This could be implicated in a regulatory loop of macrophage M2 phagocytosis. Given that hyperactive TGFβ often causes excessive macrophage phagocytic activities potentially leading to fibrotic evolution [136], the inhibition of the TGF-β-HDAC3/Smad-TIMAP pathway could represent a strategy to slow down the progression of renal fibrosis. Even in this case, it still remains to be discovered whether these mechanisms involve the activation of an EMT program.

Recent studies have also evaluated the role of HDAC8 on renal fibrosis, always belonging to class I of HDACs [137]. HDAC8 is overexpressed in UUO mice and the use of a selective inhibitor for this deacetylase, such as PCI34051, or gene silencing determines a slowdown of fibrotic progression by inhibiting the TGF-β-dependent EMT process. A very important fact is that the high expression of HDAC8 arrests renal tubular epithelial cells in the G2/M phase, determining the activation of Snail, a known pro-EMT and pro-fibrotic factor. The use of HDAC8 inhibitors leads to an inhibition of EMT and is capable of determining an increase in Klotho levels, thus acting as anti-fibrotic factors.

##### Class II Histone Deacetylases in EMT-Dependent Fibrosis

In the intertubular, extraglomerular, and extravascular spaces of the kidney, HDAC6 has been demonstrated to contribute to TGF-β-induced EMT. Although the mechanism of HDAC6 induction is not clear, this was enhanced by TGF-β, and subsequently, the activated HDAC6 deacetylated the renal tubular epithelial cytoskeletal protein (α-tubulin). α-tubulin deacetylation induces cytoskeletal rearrangement during the EMT process [138,139]. In addition, in unilateral ureteral obstruction (*UUO*) mice used as models of kidney fibrosis, HDAC1, 4, 5, 6, and 10 were overexpressed and HDAC8 was downregulated, and this seemed to determine pro-fibrotic events through a TGF-β1/Smad-independent pathway [140].

Supporting the role of histone acetylation in EMT-dependent fibrotic diseases, HDAC7 has also been demonstrated to regulate collagen deposition and other ECM protein accumulation in fibroblasts derived from patients affected by systemic sclerosis, and indeed, siRNA-mediated depletion of *HDAC7* reduced ECM production in these cells, resulting in an evident decrease in fibrogenesis [10]. However, the mechanism of action of class IIb HDACs in the fibrotic process will certainly require further investigation.

### 5.3. Histone Methylation and Demethylation Affects EMT-Dependent Fibrosis

Recently, it was demonstrated that the blocking of histone/lysine methyltransferases, such as the enhancer of zeste homolog 2 (EZH2), a histone H3 lysine 27 trimethylation methyltransferase, and SET and MYND domain-containing protein 2 (Smyd2), a histone H3 lysine 36 trimethylation methyltransferase, had a negative effect on EMT and consequently attenuated renal fibrosis [141]. However, knowledge of the role of demethylases is still limited and very recent. Lysine-specific demethylase 1 (LSD1), also known as KDM1A, is the first histone/lysine demethylase that specifically targets mono- and dimethylated H3K4 [142]. The upregulation of LSD1 was involved in renal tubular cell EMT, deposition of ECM proteins, and renal fibrosis progression in UUO model mice [143]. This was confirmed through the use of specific inhibitors or siRNA targeting LSD1, which reduced TGF-β1-induced EMT and blocked the activation of renal fibroblasts through TGF-β1/Smad3 and LSD1/PKC/α-Akt/STAT3 signaling pathways [143].

In addition, it was demonstrated that the different methylation status of specific lysines present in histones can lead to gene induction or suppression. In general, methylation of histone H3 lysine 4 (H3K4), H3K36, and H3K79 stimulates gene transcription, while H3K9, H3K27, and H4K20 methylation leads to gene suppression [144]. ZEB1 is a zinc-finger transcription factor implicated in the induction of EMT. It works on the promoter of the E-cadherin gene, suppressing its synthesis. The active constituent derived from Schisandra chinensis (SchB) appears to operate on TGF-β-mediated EMT through epigenetic inhibition of ZEB1 [145]. The ability of Sch B to enhance H3K9me3 levels of the ZEB1 promoter and to inhibit transcription of the *ZEB1* gene, which is crucial for TGF-β-induced EMT, was recently demonstrated [145]. SETDB1 (also known as ESET or KMT1E) is a specific histone methyltransferase able to block euchromatin genes by regulating the methylation of histone H3K9 [146]. SETDB1 seems to be able to trigger the trimethylation of histone H3K9 (H3K9me3) on the *Snai1* promoter region, leading to EMT reorganization, which is promoted by TGF-β-induced iron accumulation [147]. Confirming these observations, the knockdown of *SETDB1* reduced H3K9me3 and enhanced TGF-β/Snail-mediated EMT, which was accompanied by increased ferroptosis [147]. In radiation-induced lung fibrosis, Nagaraja et al. showed increased expression of H3K9me2/3 in irradiated cells [148]. This effect was reversed by the use of specific histone methyltransferase G9a inhibitor, which led to irradiated cells showing higher expression of epithelial markers and not of mesenchymal ones [148].

### 5.4. Epigenetic Involvement of ncRNAs in EMT-Related Fibrosis

NcRNAs were also recently recognized as potential key inductors of fibrogenesis [149]. Noncoding RNAs are micro-sequences of RNA transcribed but not translated into proteins, and they are grouped into short ncRNAs and long ncRNAs [150,151]. Increasing evidence has highlighted the involvement of noncoding RNAs in the EMT process.

#### 5.4.1. MiRNAs in EMT-Dependent Fibrosis

Recently, important studies have reported that miRNAs are involved as regulators or activators of signaling pathways in the induction of fibrogenesis triggered by the EMT process. To investigate the correlation between miRNAs and EMT, Liang et al. have focused their investigations on the involvement of miRNAs in pulmonary fibrosis induced by EMT. In particular, the study investigated the impact and mechanism of miR-26a in mice affected by experimental pulmonary fibrosis. It was discovered that miR-26a is significantly downregulated and modulates high-mobility group protein A2 (HMGA2), a key regulator of the EMT process [152]. Therefore, inhibition of miR-26a in lung epithelial cells determines the induction of EMT, in which the epithelial cells acquire the features of the mesenchymal phenotype and thus transform into myofibroblasts. Interestingly, the overexpression of miR-26a reduced the EMT process triggered by TGF-β in adenocarcinoma A259 cells [153]. An interesting study has evidenced that, in mice exposed to silicone to induce pulmonary fibrosis, an overexpression of miRNA let-7d led to a reduction in HMGA2 expression and inhibition of EMT; while the suppression of miRNA let-7d augmented HMGA2 expression and triggered silica-induced EMT [154].

Additionally, Wang and colleagues have demonstrated that overexpression of miR-221 diminished the expression of HMGA2, reducing the EMT events in A549 and human bronchial epithelium (HBE) cell lines [155]. Therefore, using a mouse model of bleomycin (BLM)-induced pulmonary fibrosis confirmed the effect of miR-221 on the EMT process. 

Recent experiments performed by Li and collaborators in radiation-induced pulmonary fibrosis (RIPF) have isolated extracellular vehicles (EVs) derived from mouse mesenchymal stem cells (mMSC-Exos) and examined their effects both in vitro and in vivo. The results obtained showed that mMSC-Exos protect cells and reverse the EMT process induced by radiation.

Interestingly and correlated with these data is the finding that miR-466f-3p in de-534 EVs separated from mMSC-Exos inhibited radiation-induced evolution by inhibiting AKT/GSK3β through c-MET [156].

In recent years, an interesting study conducted by Wang et al., using high-throughput sequencing, has shown that miR-155–5p is significantly downregulated in RIPF and acts as a key regulator of RIPF via the GSK-3β/NF-κB pathway. Blocking glycogen synthase kinase-3β (GSK-3β), a functional target of miR-155–5p, reversed radiation-induced EMT through the NF-κB pathway, preventing the progression of RIPF [157]. This suggests that ectopic expression of miR-155–5p reduces RIPF in mice through the GSK-3β/NF-κB pathway. These data were confirmed by recent findings in which it was demonstrated that ionizing radiation (IR) impeded the transcription of miR-486–3p, which determines the activation of EMT target genes such as *Snail*, leading to the induction of EMT in radiation-induced pulmonary fibrosis [158].

In addition, Liang et al. have demonstrated that miR-541-5p repression through Myeloid Zinc Finger 1 (MZF1) factor, an important transcriptional repressor, triggers the EMT process induced by irradiation, contributing to RIPF. In particular, miR-541-5p modulates the effect of the *Slug* gene on the EMT process. Irradiation activates MZF1 to downregulate miR-541-5p in alveolar epithelial cells, and hence induces EMT, contributing to RIPF by targeting *Slug* [158].

Therefore, using bioinformatics analysis, it was found that Fos-related antigen 1 (Fra-1) is a potential target of miR-34c-5p. The miR-34c-5p/Fra-1 axis represses the induction of the PTEN/PI3K/AKT signaling pathway and inhibits the EMT process [159]. This result can provide a promising therapeutic approach to alleviate the progression of pulmonary fibrosis. Recent studies have highlighted the role of miR-21 as a profibrotic factor since its upregulation was noted in patients with severe fibrosis kidney disease. MiR-21 influences the expression of different metalloproteinases (MMPs) through the downregulation of PTEN during TGF-β1-promoting EMT [160]. Furthermore, the inhibition of miR-21 reduces the induction of profibrotic genes in human podocytes and tubular cells in renal diseases [161,162]. Therefore, miR-21 modulates the TGF-β pathway, but TGF-β1 can also induce the expression of miR-21 through Smad signaling [163]. Microarray analysis has evidenced that upregulation of miR-21 in tubular epithelial cells depends on activation of TGF-β/Smad3 signaling. These data were confirmed in normal and Smad3 knockdown tubular epithelial cells wherein the suppression of Smad3, but not of Smad2, prevented cells from upregulating miR-21 in response to TGF-β [163,164]. Upregulation of miR-21 involves different mechanisms in promoting renal fibrosis, such as the decrease in dimethylarginine dimethylaminohydrolase 1 (DDAH1) expression. This leads to an elevated level of asymmetric dimethylarginine (ADMA), which is an endogenous repressor of nitric oxide synthase (NOS), thus diminishing the production of NO [165]. Emerging evidence has revealed an elevated expression of miR-34a in renal fibrosis. miR-34a directly links to the 3′ UTR of Klotho mRNA, leading to the downregulation of Klotho that preserves the kidneys against severe and chronic failure, thus contributing to renal fibrosis [165,166,167]. The overexpression of miR-34a-5p in the kidney tissues of patients suffering from type 2 diabetes mellitus and mice affected by diabetic nephropathy (DN) was associated with Ski-related novel gene (*SnoN*) downregulation and, thus, with the activation of EMT-dependent fibrosis [168]. MiR-130a-3p plays a pro-fibrotic role; indeed, it is implicated in the modulation of EMT and provokes severe TGF-β1-induced fibrosis in renal tubular epithelial cells [169]. Furthermore, the repression of miR-130a-3p was found to be linked with a marked reduction in α-SMA and vimentin, as well as an increase in levels of E-cadherin expression. Moreover, in vitro studies have evidenced the central role of miR-130a-3p in the modulation of Smads, which are implicated in the TGF-β signaling pathway in the course of renal fibrosis evolution [169].

Another study conducted by Bai et al. has evidenced that the overexpression of miR-27b-3p significantly reduced TGF-β1-dependent EMT through the decrease in mesenchymal factors in cell cultures [170]. These findings evidenced that augmented expression of miR-27b-3p avoids renal fibrosis through EMT suppression. Therefore, overexpression of miR-27b-3p diminished the expression of p-STAT1 and STAT1 [170], promoting renal fibrosis induced by TGF-β1. Moreover, both apoptosis and the EMT process were shown to be involved in miR-27b-3p-mediated control of renal fibrosis. In addition, the overexpression of miR-27b-3p repressed TGF-β1-induced and Fas-mediated apoptosis in human kidney cells through the downregulation of pro-apoptotic factors such as active caspase 3, Fas, and active caspase 8 [170].

MiR-30e expression is downregulated in diabetic nephropathy patients, and this event is linked with an augmented EMT process in renal tubule epithelial cells. In turn, the upregulation of this miRNA determines low levels of mesenchymal markers such as collagen I and vimentin and elevated levels of E-cadherin expression. These findings demonstrate that miR-30e plays an antifibrotic role and has protective activities [171].

The role of the miR-200 family in renal fibrosis is conflicting. It has been demonstrated that miR-200 may play an antifibrotic role [172], and in fact, findings confirmed that miR-200b suppresses TGF-β1-induced EMT through the reduction in fibronectin and the increase in E-cadherin [173]. The suppression of the TGF-β1-induced EMT pathway was demonstrated to be independent of TGF-β1-induced p-Smad2/3, phospho-p38, and p42/44 signaling. miR-200a is, furthermore, able to repress TGF-β2 expression, thus strongly reducing the evolution of renal fibrosis [173].

Emerging evidence has highlighted the profibrotic role of miR-32 in liver fibrosis. Indeed, hepatic fibrosis induced by the EMT process and influenced by MTA3 expression in hyperglycemic conditions was inhibited by anti-miR-32. These results demonstrated that miR-32 knockdown may ameliorate liver fibrosis progression, suggesting possible targets for the treatment of liver fibrosis [174].

An interesting study has evaluated the effect of miR-451 in diabetic cardiomyopathy influenced by endothelial to mesenchymal transition (EndMT). Growing evidence indicates that miR-451 silencing inhibits the EndMT process in diabetic mice hearts. This study also showed the effect of miR-451 knockdown on the reduction of hyperglycemia-induced EndMT in mouse cardiac endothelial cells, which led to an evident decrease in the progression of cardiac fibrosis [175].

#### 5.4.2. CircRNA Regulation of Fibrosis Correlated to EMT

CircRNAs are RNA molecules with a loop structure primarily produced through back-splicing of pre-mRNA [176]. Most studies on non-coding RNAs have pointed to miRNAs and lncRNAs; however, with progress in bioinformatics analysis and the availability of advanced RNA-sequencing technologies, research has moved towards circRNAs. Several findings have shown that circRNAs participate in various physiological and pathological mechanisms through different processes [176]. Several studies have demonstrated that circRNAs play a regulatory role in EMT-dependent pulmonary fibrosis. In an experimental evaluation in which lung fibrosis was induced by silica, upregulation of the circRNA CDR1as served as a sponge and inhibitor for miR-7, leading to the release of TGFBR2 and promoting EMT [177]. Recently, Li et al. have demonstrated an elevated overexpression of the hsa_circ_0044226 in IPF tissue [178]. Data from Qi and collaborators supported the hypothesis that the downregulation of circRNAs has an antifibrotic effect, demonstrating that the reduced expression of hsa_circ_0044226 attenuates pulmonary fibrosis through the inhibition of *CDC27* expression, a parental gene for hsa_circ_0044226 [179]. New findings in this context have demonstrated that blocking circZC3H4 effectively inhibits the EMT process and blocks the progression of pulmonary fibrosis induced by SiO_2_ [180].

Furthermore, circZC3H4 functions as a sponge for miR-212, modulating ZC3H4 expression and consequently inducing the EMT process [181]. These findings demonstrate the important role of circRNAs in regulating EMT-associated pulmonary fibrosis, even if further investigations and discoveries in this field are requested to fill these knowledge gaps (Figure 3).

#### 5.4.3. LncRNA Regulation of EMT Related to Fibrosis

Long non-coding RNAs (lncRNAs) represent a class of non-coding transcribed RNA molecules of more than 200 nucleotides [182]. Accumulating evidence has highlighted the key role of lncRNAs in the control of EMT. Furthermore, several lncRNAs act as competitive endogenous RNAs (ceRNAs), preventing the link of miRNA to its target genes [183].

Interestingly, a recent study showed that, during the EMT process of lung epithelial cells, elevated levels of long non-coding RNA (lncRNA)-ATB were detected, lncRNA activated by TGF-β, which may lead to decreased levels of miR-200c. The inhibitory role of lncRNA-ATB on miR-200c was also demonstrated in silica-induced EMT-dependent pulmonary fibrosis [184]. Furthermore, it has been demonstrated that miR-200c is able to target ZEB1, to enhance E-cadherin levels, and subsequently inhibit the EMT process, thus potentially ameliorating silicosis [184].

Xu et al. (2021) have demonstrated that lncRNA-ATB has pro-fibrotic effects by downregulating miR-29b-2-5p and miR-34c-3p in vitro. This effect was supported by the observation that the overexpression of miR-29b-2-5p or miR-34c-3p inhibits the pro-fibrotic effects of lncRNA-ATB and thus attenuates the induction of EMT [185].

Sun et al. identified a large number of upregulated and downregulated long non-coding RNAs (lncRNAs) in lung tissue induced by paraquat (1,1-dimethyl-4,4-bipyridyl dichloride), a herbicide that can be fatal to humans and, in particular, can induce pulmonary fibrosis. The authors identified Zeb2 and Hoxa3, modulators of EMT, as target genes of two upregulated lncRNAs: uc.77 and 2700086A05Rik. These findings underline the crucial role of lncRNAs in the regulation of EMT during lung fibrosis [186].

Wang et al. (2022) discovered that the lncRNA miR99AHG functions as a competitive endogenous RNA (ceRNA) with miR-136–5p. As a consequence, ubiquitin-specific protease 4 (USP4) is less degraded by miR-136-5p. This determines the regulation of the expression of angiotensin-converting enzyme 2 (ACE2), a downstream target gene of USP4, and activation of the EMT process in the alveolar epithelial cells type II [187].

Zhan and collaborators have investigated the role of lncRNA MEG3 in a rat model of lung fibrosis induced by nickel oxide nanoparticles (NiO NPs) via activating TGF-β1, which was associated with the activation of the EMT process. The study showed that lncRNA MEG3 suppressed the TGF-β1 pathway, the EMT process, and collagen accumulation [188].

Intriguingly, findings have shown that the lncRNA ZEB1 antisense RNA 1 (ZEB1-AS1) was upregulated in a rat model of BLM-induced pulmonary fibrosis and in TGF-β1-induced alveolar type II epithelial (RLE-6TN) cells. Silencing of *ZEB1-AS1* inhibited the induction of EMT and reduced BLM-induced fibrogenesis [189].

Several other lncRNAs can be involved in liver fibrosis. In particular, GAS5 acts as a sponge platform for miR-23a through the PTEN/PI3K/Akt pathway, which could slow down the progression of hepatic fibrosis [190]. Chen and collaborators have discovered that Meg8 lncRNA is overexpressed in activated HSC, injured hepatocytes, and fibrotic livers. Furthermore, knockdown of Meg8 significantly inhibited the expression of epithelial factors and induced the expression of mesenchymal markers in hepatocytes [191]. (Figure 3). All of the data reported and related to epigenetic involvement of ncRNAs in EMT-related fibrosis are summarized in Table 1.

## 6. Conclusions and Future Perspective

This article represents an attempt to collect relevant data on the role of epigenetics in regulating EMP/EMT-dependent fibrosis. The purpose is to arouse growing interest in the scientific world and clarify the molecular mechanisms underlying this regulation of gene expression. This could identify factors predisposed to the onset of diseases related to the triggering of the EMP/EMT program, such as cancer or inflammatory diseases. After decades of studies, EMT is now considered a key physiological process active in embryo genesis and a pathological mechanism triggered in fibrotic diseases and cancer. Furthermore, recently, the meaning of EMT has also been revised by scientists, who have identified various degrees of cellular transformation toward a mesenchymal phenotype, so much so that they have coined the term EMP, underscoring cellular plasticity and the ability of an epithelial cell to assume intermediate phenotypes. Recent studies have unveiled the role of epigenetics in the control of EMP/EMT dynamics and, in general, in cellular plasticity. Several epigenetic modifications have been identified as capable of modifying gene transcription during the process of EMP/EMT related to fibrogenesis, adding even more complexity to the process of EMP/EMT-dependent fibrosis, which is finely regulated by various molecularly interconnected mechanisms. We hope that having summarized in this review the most recent discoveries in this very innovative field will provide new perspectives on molecular aspects and therapeutic approaches intended to regulate and reverse the EMP/EMT-dependent fibrotic process.

## Figures and Tables

**Table 1:** microRNA regulation of EMT-dependent fibrosis.

ncRNAs	Target	Action Modes	Outcomes	References
miR-26a	*HMGA2*	Downregulation of miR-26a promotes EMT process	Pulmonary fibrosis	[152]
miRNA let-7d	reduces *HMGA2* expression	Overexpression of miRNA let-7d inhibits EMT process	Silicone-induced lung fibrosis	[154]
miR-221	reduces *HMGA2* expression	Overexpression of miR-221 inhibits EMT process	Bleomycin (BLM)-induced pulmonary fibrosis	[155]
miR-466f-3p	mMSCs-exo	Antifibrotic features of miR-466f-3p that prevent radiation-induced EMT	Radiation-induced pulmonary fibrosis	[156]
miR-155–5p	*GSK-3β*	Downregulation of miR-155–5p in radiation-induced pulmonary fibrosis	Radiation-induced pulmonary fibrosis	[157]
mir-486–3p	*Snail* gene	Downregulation of mir-486–3p promotes EMT process	Radiation-induced pulmonary fibrosis	[158]
miR-541-5p	*MZF1, Slug* gene	miR-541-5p repression induces EMT process	Radiation-induced pulmonary fibrosis	[158]
miR-34c-5p	*Fra-1*	miR-34c-5p/Fra-1 axis represses activation of EMT process	Pulmonary fibrosis	[159]
miR-21	MMPs, activation of TGF-β/Smad3 signaling	Upregulation of miR-21 induces TGF-β1, promoting EMT	Kidney fibrosis	[160,163,164]
miR-21	*DDAH1*	Upregulation of miR-21 decreases DDAH1 and increases ADMA, diminishing the production of NO	Kidney fibrosis	[165]
miR-34a	3′ UTR of Klotho mRNA	Increased expression of miR-34a downregulates Kloto	Kidney fibrosis	[154]
miR-34a-5p	*SnoN*	Overexpression of miR-34a-5p determines the downregulation of SnoN and induction of EMT	Kidney fibrosis	[168]
miR-130a-3p	*Smads*	Downregulation of miR-130a-3p modulates Smads, induction of EMT	Kidney fibrosis	[169]
miR-27b-3p	α-SMA, fibronectin, collagen III, vimentin	Overexpression of miR-27b-3p reduces EMT process. Overexpression of miR-27b-3p reduces p-STAT1 and STAT1 induces EMT.	Kidney fibrosis	[170]
miR-30e		Downregulation of miR-30e expression in diabetic nephropathy, increases EMT	Kidney fibrosis	[171]
miR-200b		miR-200b inhibits TGF-β1-induced EMT, ameliorates renal fibrosis	Kidney fibrosis	[172]
miR200a		miR-200a represses TGF-β2 expression, inhibiting the development of renal fibrosis	Kidney fibrosis	[172,173]
miR-32	*MTA3*	Profibrotic role of miR-32	Liver fibrosis	[174]
miR-451		Induced by EndMT	Cardiac fibrosis	[175]
CDR1	sponge for miR-7	Upregulation of CDR1 promotes EMT process	Silica-induced pulmonary fibrosis	[177]
hsa_circ_0044226	*CDC27*	Upregulation of hsa_circ_0044226 promotes EMT	Idiopatic pulmonary fibrosis	[178]
circZC3H4	sponge for miR-212	Modulation of ZC3H4 expression induces EMT process	Idiopatic pulmonary fibrosis	[181]
lncRNA-ATB	sponge for miR-200c	lncRNA-ATB promotes EMT program	Silica-induced pulmonary fibrosis	[184]
uc.77 and 2700086A05Rik	*Zeb2* and *Hoxa3*	Upregulation of uc.77 and 2700086A05Rik induces EMT process	Pulmonary fibrosis	[186]
lncRNA miR99AHG	miR-136–5p	miR99AHG regulates the expression of ACE2, a target gene of USP4, and induces EMT process	Pulmonary fibrosis	[187]
lncRNA MEG3		MEG3 induces NiO NPs via activating TGF-β1 and activation of EMT process	Pulmonary fibrosis	[188]
ZEB1-AS1		Upregulation of EB1-AS1 induces EMT process	Pulmonary fibrosis	[189]
GAS5	sponge for miR-23a	Activation of PTEN/PI3K/Akt pathway induces EMT process	Hepatic fibrosis	[190]
Meg8		Overepression of Meg8 promotes EMT process	Hepatic fibrosis	[191]

## Data Availability

Not applicable.
